# Mental health experiences of healthcare professionals during COVID-19

**DOI:** 10.4102/sajip.v47i0.1865

**Published:** 2021-09-28

**Authors:** Lindsay J. Cook, Tasneem Hassem, Sumaya Laher, Tarique Variava, Enid Schutte

**Affiliations:** 1 Department of Psychology, Faculty of Humanities, University of the Witwatersrand, Johannesburg, South Africa

**Keywords:** COVID-19, mental health, healthcare workers, resilience, coping styles, social support, anxiety, depression

## Abstract

**Orientation:**

The coronavirus disease 2019 (COVID-19) pandemic led to fundamental changes in the workplace for many, particularly healthcare workers.

**Research purpose:**

This study explored healthcare workers’ (ophthalmologists, nurses and support staff) experiences of anxiety, depression, burnout, resilience and coping strategies during lockdown Levels 2 and 3 in an Ophthalmic consulting practice and hospital in South Africa.

**Motivation for the study:**

The increased workplace stress and vulnerability associated with working during the COVID-19 pandemic introduced an unprecedented level of risk for healthcare workers. Factors contributing to psychological distress must be identified and appropriately mitigated, to prevent dire human and economic costs.

**Research approach/design and method:**

A survey was sent out at two separate times to a convenience sample of 31 and 15 healthcare workers respectively. The survey consisted of a demographics section, Hospital Anxiety and Depression Scale, Burnout Measure short-version, Brief Cope Inventory, Connor Davidson Resilience Inventory and six open-ended questions investigating personal health and support experiences during COVID-19. Descriptive analyses and thematic analysis were used for data analysis.

**Main findings:**

The sample of healthcare workers experienced some degree of psychological distress, including anxiety, burnout and a lack of social support on both surveys. However, these symptoms were alleviated by personal factors, including positive coping mechanisms, high resilience and organisational support.

**Practical/managerial implications:**

Healthcare facilities should consider in-house structures focusing on building resilience and positive coping mechanisms, whilst ensuring that workplace conditions are optimal for staff members.

**Contribution/value-add:**

This study provides some insight into both the risk and protective factors experienced by health workers during the COVID-19 pandemic.

## Introduction

### Orientation

On 30 January 2020, the coronavirus disease 2019 (COVID-19) pandemic was declared as a public health emergency of international concern (World Health Organization, 2020). By 22 December 2020, the death rate was over 1.3 million worldwide, making this the major global health crisis of this century, which has and will continue to have devastating social, economic and political consequences (World Health Organization, 2020). Globally, healthcare workers have been declared as essential workers in the fight against the COVID-19 pandemic. However, the scale of this pandemic has led to unique and unprecedented workplace considerations for this group.

### Research purpose and objectives

This study aims to contribute to an understanding of the psychological states, resilience and coping mechanisms of South African healthcare workers during the COVID-19 pandemic.

### Literature review

In an attempt to curb the spread of the coronavirus, extreme measures have been imposed globally, including restricting travel, quarantining citizens and social distancing measures. With this regard, South Africa is no exception, with the country experiencing an extensive countrywide lockdown from the 23 March to 23 April 2020 (South African Government, 2020). Lockdown Level 5 led to the closing of all non-essential businesses, with their employees being encouraged to work from home. However, healthcare workers (HCWs) were deemed a vitally important resource in effectively responding to COVID-19 and were required to report to work during all levels of lockdown, from Level 5 through to the current Level 1 (Robertson, Maposa, Somaroo, & Johnson, 2020).

Whilst the literature primarily describes HCWs as healthcare professionals (doctor, nurse, psychologist, etc.), for the purposes of this study, ‘healthcare worker’ is broadly defined to include anyone involved in care and/or healthcare work, for example, health professionals, student clinicians, cleaners and receptionists. All healthcare staff are at risk and as such, it is necessary to understand the challenges experienced by all in this context and the subsequent effect that this may have on their work performance.

## Mental health and performance

Prior to the COVID-19 pandemic, the South African healthcare system was burdened and under severe strain as a result of the lack of resources, personnel and critical facilities, as well as the considerable number of patients living with human immunodeficiency virus (HIV), tuberculosis, malnutrition and diabetes (George, Quinlan, Reardon, & Aguilera, 2012). As a middle-income country, South Africa should have a ratio of 180 doctors per 100 000 people; however, it is estimated that the actual doctor to patient ratio is 62 per 100 000 people (Econex, 2015). The lack of personnel in hospitals has a ripple effect on the staff, as highlighted by Rispel (2015), who found that over 60% of nurses were too tired to work effectively whilst on duty, as a result of continual overtime work. Furthermore, the poor infrastructure associated with the South African healthcare system places impossible caseloads on HCWs, leading to negative patient outcomes and safety concerns because of breaches in infection control (Robertson et al., 2020). Thus, continual elevated stress, anxiety and exhaustion occur as a result of systemic challenges and their impact on HCW’s mental health, which may compromise their work performance, increase their likelihood of error and place them at higher risk of burnout and illness (Gray et al., 2019). The pandemic has exacerbated these crises. Consequently, there is an increased risk of psychological difficulties, including anxiety, depression, fear, distress, poor coping mechanisms and insomnia amongst HCWs (Robertson et al., 2020; Shaukat, Ali, & Razzak, 2020). Thus, protecting HCW’s mental health and well-being is essential during the COVID-19 pandemic.

## Risk factors for healthcare workers

With the continued spread of the virus globally, patients, HCWs and the general public around the world are facing unprecedented psychological stress, as well as pressure to adapt to new working conditions (Benítez et al., 2020). Research conducted in 34 hospitals in China measuring psychological reactions of healthcare workers during the COVID-19 outbreak reported high rates of depression (50%), anxiety (42%), insomnia (34%) and distress (72%) (Lai et al., 2020). Similarly, in a systematic review of the global literature surrounding healthcare worker’s mental health, rates of depression, anxiety and post-traumatic stress disorder (PTSD) ranged from 8.9% to 50.45%, 10.4% to 44.6% and 32% to 71.5%, respectively (Roberston et al., 2020). It is clear that HCWs are physically and psychologically challenged in ensuring the provision of high-quality care for patients (Lai et al., 2020). Similar research has been conducted in South Africa; however, it is difficult to report on the prevalence of mental health conditions because of wide ranges in different study populations, study sites and screening tools (Roberston et al., 2020).

Contributing factors to HCW’s psychological stress include working with a poor understanding of the virus and new and frequently changing protocols, increased use of personal protective equipment (PPE), prolonged working hours and inadequate hospital equipment (George et al., 2012; Walton, Murray, & Christian, 2020). For instance, the use of PPE has been shown to affect surgeons’ non-technical skills (such as communication), augment fatigue, give rise to headaches and affect their performance (Benítez et al., 2020; Ong et al., 2020). Common issues included goggle fogging leading to impaired visibility, psychomotor skill and impaired performance of manual tasks (Benítez et al., 2020). Furthermore, PPE use and hazardous working conditions generated during the pandemic cause increased fatigue during surgical procedures and jeopardises surgical judgement (Benítez et al., 2020).

Healthcare professionals appear to experience great psychological stress whilst at work but are also faced with social isolation and quarantine measures when at home. This represents another contributing factor to HCW’s psychological stress (Chersich et al., 2020). Many healthcare workers have chosen to separate themselves from their families to protect them and prevent the spreading of the virus (Chersich et al., 2020). These workers may experience stigmatisation, loneliness, or loss of trust in their communities (Chersich et al., 2020). This can be problematic as low support is an established risk factor for mental health problems for people in crises (Naushad et al., 2019). Low social support is likely to negatively impact work performance of HCWs (Saeng, Chi-Keun, & Kyu, 2020).

## Protective factors

Despite these challenges, there is evidence that positive coping skills, organisational support and resilience may mitigate the risk of burnout and PTSD (Howlett et al., 2015; Man et al., 2020; Matheson et al., 2016; Streb, Häller, & Michael, 2014). According to Labrague and De los Santos (2020), resilient nurses who perceived higher levels of organisational and social support reported lower COVID-19 related anxiety. Moreover, social support and positive coping skills reduced medical staff’s anxiety (Zhu, Wei, Meng, & Li, 2020). Such positive coping skills and resilience may contribute to the maintenance of acceptable levels of workplace performance and accuracy, which is beneficial for the safety of the HCW, the patient and the general population (Gray, 2019).

## Research questions

The following questions were considered to conduct this study:

1. What are the levels of depression, anxiety, burnout, resilience and coping mechanisms amongst healthcare workers actively working during the COVID-19 pandemic?
2. What are the qualitative, lived experiences of healthcare workers actively working during COVID-19?

## Research approach

The study followed a longitudinal, non-experimental research design, where staff members from the East London Eye Centre completed an online questionnaire via Survey Monkey. The first survey commenced on 01 June 2020 and closed on 31 July 2020. The follow-up survey occurred on 10 August 2020 and closed on 20 September 2020, which corresponded with the lockdown Levels 3 and 2 in South Africa.

## Research method

### Research participants

This study was conducted in a private, urban ophthalmic clinic in East London, which caters for much of the rural population in the Eastern Cape. A non-probability convenience sample of 31 of the available 50 staff members of the Eye Centre participated in the first survey and 15 staff members participated in the second survey (see Table 1 for demographic variables). Majority of the participants in time 1 and 2 identified as being female (*n* = 22, 78.6%; *n* = 11, 73.3, respectively), Christian (*n* = 26, 83.9%; *n* = 13, 86.7%) and they spoke English as a home language (*n* = 15, 53.6%; *n* = 10, 66.7%, respectively). The lowest level of qualification for participants in time 1 was a Matriculation (*n* = 5, 33%), whilst in time 2, it was an undergraduate degree or diploma degree (*n* = 5, 33.3%). Majority of participants in surveys 1 and 2 were in a relationship (*n* = 16, 53.3%, *n* = 10, 66.7%, respectively) or married (*n* = 16, 51.6%, *n* = 9, 60%). In both time 1 and 2, the majority of participants had no chronic physical or mental illness (*n* = 20, 64.5%, *n* = 11, 73.3%). Whilst the majority of participants in time 1 had two children (*n* = 9, 26%), the majority of participants in time 2 had no or two children (*n* = 4, 26.6%, *n* = 4, 26.6%). Most of the participants in time 1 reported living with a partner and child (*n* = 11, 35.5%), whilst in time 2, most participants reported living alone (*n* = 6, 40%). The mean age for participants in time 1 was 40.68 years (standard deviation [SD] = 10.140) with individuals reporting working between 0 and 34 years at the site (*M* = 7.75, SD = 8.97) and 1 to 36 years of work experience since graduating (*M* = 17.193, SD=10.47). The mean age for participants in time 2 was 43.6 years (SD = 10.888), with individuals reporting having worked at the site between 1 and 23 years (*M* = 11.38, SD = 9.288) and had between 7 to 36 years of work experience since graduating (*M* = 22.40, SD = 10.658). Participants in time 1 included cleaners (*n* = 2), managers (*n* = 4), ophthalmologists (*n* = 3), optometrists (*n* = 2), general doctors (*n* = 1), nurses (*n* = 9), as well as general workers (*n* = 10). Whereas, in time 2, the participants included ophthalmologists (*n* = 4), optometrists (*n* = 2), nurses (*n* = 4) and general workers (*n* = 5).

**TABLE 1:** Demographic variables for time 1 and 2.

Demographics	Variable	Time 1		Time 2	
		Frequency	Percentage	Frequency	Percentage
Gender†	Female	22	78.6	11	73.3
	Male	6	21.4	4	26.7
Home language‡	Afrikaans	9	32.1	5	33.3
	English	15	53.6	10	66.7
	IsiXhosa	2	7.1	-	-
	Setswana	2	7.1	-	-
Highest level of education	Other	2	6.7	-	-
	Pre-school	1	3.3	-	-
	Primary school	1	3.3	-	-
	Some high school	2	6.7	-	-
	Matric	5	33.3	3	20.0
	Undergraduate degree or diploma	7	23.3	5	33.3
	Honours	4	13.3	3	20.0
	Master’s	3	10.0	4	26.7
Religious affiliation	Other	2	6.5	-	-
	No religion	3	9.7	2	13.3
	Christianity	26	83.9	13	86.7
Number of children	0	7	22.6	4	26.6
	1	6	19.4	3	20.0
	2	9	29.0	4	26.6
	3	5	16.1	3	20.0
	4	4	12.9	1	6.7
Relationship status§	Yes	16	53.3	10	66.7
	No	14	46.7	5	33.3
Marital status	Yes	16	51.6	9	60.0
	No	14	48.4	6	40.0
Chronic condition	Yes	10	33.3	4	26.7
	No	20	66.7	11	73.3
Chronic medication	Yes	11	35.5	4	26.7
	No	20	64.5	11	73.3
Living condition	Alone	3	9.7	1	6.7
	With a partner	6	19.4	6	40.0
	With a partner and child	11	35.5	5	33.3
	With children	4	12.9	2	13.3
	With immediate family	6	19.4	1	6.7
	With other relatives	1	3.2	-	-

Note: *N* = 31 for time 1, except where indicated otherwise: †, *N* = 28; ‡, *N* = 30; §, *N* = 15 for time 2.

## Measuring instruments

The online questionnaire consisted of three sections, namely, demographic, mental health screening instruments as well as open-ended questions. The following demographic variables were requested from the participants: age, gender, home language, highest level of education, current occupation, chronic psychical or mental health condition, chronic medication, marital status, relationship status, place of professional training, number of years practising at the site as well as number of years practising since graduating.

### Hospital Anxiety and Depression Scale

Depression and anxiety were measured using the Hospital Anxiety and Depression Scale (HADS). The HADS has two subscales, namely, anxiety and depression. Both subscales consisted of seven items each and a four-point response-format. Each item had a unique anchor (0–3). The HADS has been validated on a sample of HIV and/or acquired immune deficiency syndrome (AIDS) patients in South Africa (insert ref). The internal consistency reliability coefficients for the anxiety and depression subscales were excellent at 0.86 and 0.81, respectively. The HADS has been validated in a community setting (Snaith, 2003).

### Burnout Measure – Short Version

Burnout was measured utilising the Burnout Measure – Short Version (BMS-S). The measure consists of 10 items which assess the frequency of experiencing symptoms of mental, emotional as well as physical exhaustion. Participants were required to respond to the items using a seven-point Likert scale (*1* = never, *7* = always) in relation to how they felt about their work. Fatoki (2019; as cited in Malach-Pines, 2005) reported an alpha coefficient of 0.82 in the South African context. For this study, an excellent reliability coefficient of 0.91 was achieved.

### Brief COPE Inventory

Coping skills were assessed using the brief COPE inventory, which consists of 28 items, and 14 subscales, namely, Self-Distraction, Active Coping, Denial, Substance Use, Use of Emotional Support, Use of Informational Support, Behavioural Disengagement, Venting, Positive Reframing, Planning, Humour, Acceptance, Religion and Self-Blame. Each subscale consists of two items. A four-point Likert scale response format was used for participants to rate how they utilised a particular coping mechanism during a stressful situation. As a result of this study being conducted during lockdown Level 4, item 19 relating to going to the movies was removed. Kotzé, Visser, Makin, Sikkema and Forsyth (2013) reported an alpha coefficient of 0.63 for the tool. For this study, the internal consistency reliabilities for the coping subscales ranged between 0.63 and 0.91, with the exception of five subscales, namely, Self-distraction (α = 0.56), Venting (α = 0.32), Positive framing (α = 0.50), Humour (α = 0.48) and Self-blame (α = 0.45).

### The Connor-Davidson Resilience Scale

Resilience was measured using the CD-RISC-10 (Connor-Davidson Resilience Scale) (Campbell-Sills & Stein, 2007), which assesses an individual’s ability to bounce back after experiencing trauma, tragedy or stressful events (Connor & Davidson, 2003). The response format for the CD-RISC-10 is a 5-point Likert type scale, ranging from not at all (0) to true nearly all of the time (4). A maximum score that can be obtained on the instrument is 40. Within a South African adolescent population, a reliability coefficient of 0.93 was established (Jørgensen & Seedat, 2008; Vaishnavi, Connor, & Davidson, 2007). An internal consistency reliability coefficient of 0.91 was achieved for this study.

### Self-report, open-ended questions

Six open-ended questions were presented to the participants to gain a more in-depth understanding of their lived experiences of working during the COVID-19 pandemic. The questions were concerned with the participants’ experience of work as the COVID-19 outbreak began in South Africa, their general health during this time and their support mechanisms at home and at work. The last question asked what the participant would tell the Minister of Health, if given the opportunity.

## Research procedure

The surveys were distributed online as well as in hard copy for the convenience of the staff members who did not have access to the internet or a computer. Initial contact with participants was initiated via an email disseminated to both consulting and hospital staff. The email gave a brief overview of the study and offered a link to a SurveyMonkey survey. Once informed consent was given, the participants had access to the survey. Participation was anonymous unless participants provided contact information for a follow-up interview. Lastly, the participants were provided with details of free telephonic counselling if they experienced any distress whilst completing the survey. The survey comprised three sections, which took approximately 20–25 min to complete. Once the data were collected, they were analysed and coded by one of the researchers and the appropriate analyses were conducted.

### Statistical analysis

Quantitative data were analysed using SPSS Version 27 (IBM Corp, 2019). For nominal demographic variables, frequencies and percentages were calculated. Means, SDs, minimum and maximum values as well as skewness coefficients were calculated for all the mental health instruments used. Because of the low response rate in the second survey, only nine participants who participated in the first survey could be matched with their data in the second survey and thus no comparative statistical analyses were computed. Qualitative data were analysed using thematic analysis as specified by Braun and Clark (2006).

### Ethical considerations

The study was approved by Human Ethics Committee (Medical) at the University of the Witwatersrand (clearance number: M200461) and permission was granted by the board of the clinic. Surveys were distributed online as well as in hard copy to help the staff members who did not have access to the internet or a computer. Initial contact with the participants was initiated via an email disseminated to both consulting and hospital staff.

The email gave a brief overview of the study and offered a link to a SurveyMonkey survey. Once informed consent was given, the participants had access to the survey. The survey comprised three sections, which took approximately 20–25 min to complete. Once the data were collected, they were analysed and coded by one of the researchers and the appropriate analyses were conducted. Participation was anonymous unless participants provided contact information for a follow-up interview. Lastly, the participants were provided with details of free telephonic counselling if they experienced any distress whilst completing the survey.

## Results

### What are the levels of physical and psychological health, resilience and coping amongst healthcare workers actively working during the COVID-19 pandemic?

Overall, as evidenced, healthcare workers at the East London clinic present with good psychological health (Table 2). For the HADS anxiety and depression scales, the mean scores obtained by participants do not indicate a prevalence of any mood disorder. During time 1, the most commonly reported coping mechanisms endorsed by participants were active coping (*M* = 4.00, SD = 1.633), planning (*M* = 3.742, SD = 1.673) and religion (*M* = 3.581, SD = 2.078), whilst participants in time 2 reported acceptance (*M* = 4.600, SD = 1.121), positive framing (*M* = 3.733, SD = 2.951) and active coping (*M* = 3.267, SD = 1.831) as the most commonly utilised coping mechanisms. Behavioural disengagement and substance use were the least reported coping styles used by participants in time 1 and 2. Lastly, both the resilience and burnout scores obtained during time 1 and time 2 fell within the expected range of scores.

**TABLE 2:** Descriptive statistics for time 1 and 2.

Mental health variable	Time 1					Time 2				
	*N*	Minimum	Maximum	Mean	SD	*N*	Minimum	Maximum	Mean	SD
**HADS anxiety subscale**	31	0	17	7.678	4.423	15	1.00	13.00	5.887	3.226
HADS depression subscale	31	0	14	5.355	3.937	15	0.00	12.00	4.667	3.716
**Coping styles**										
Self-distraction	31	0	6	3.129	1.839	15	1.00	6.00	2.800	1.613
Active coping	31	1	6	4.000	1.633	15	0.00	6.00	3.267	1.831
Denial	31	0	6	1.226	1.309	15	0.00	5.00	0.867	1.457
Substance use	31	0	6	0.903	1.660	15	0.00	6.00	0.533	1.552
Use of emotional support	31	0	6	2.742	1.861	15	0.00	5.00	2.733	1.792
Use of instrumental support	31	0	6	3.000	1.862	15	0.00	5.00	2.600	1.454
Behavioural disengagement	31	0	6	1.032	1.581	15	0.00	4.00	0.467	1.060
Venting	31	0	6	2.613	1.498	15	0.00	4.00	1.800	1.265
Positive reframing	31	0	6	3.355	1.664	15	0.00	6.00	3.733	2.052
Planning	31	0	6	3.742	1.673	15	0.00	6.00	3.267	2.052
Humour	31	0	6	1.677	1.620	15	0.00	5.00	1.933	1.981
Acceptance	31	0	6	4.387	1.606	15	2.00	6.00	4.600	1.121
Religion	31	0	6	3.581	2.078	15	0.00	6.00	3.400	1.920
Self-blame	31	0	6	1.323	1.514	15	0.00	4.00	0.9333	1.100
**Total resilience score**	30	9	40	26.733	8.820	14	15.00	39.00	30.000	6.680
**Total burnout score**	30	11	48	27.90	10.142	15	12.00	42.00	25.600	8.492

HADS, Hospital Anxiety and Depression Scale; SD, standard deviation.

### What are the qualitative, lived experiences of healthcare workers actively working during COVID-19 pandemic?

Overall, the qualitative data highlighted that the majority of healthcare workers experienced anxiety because of personal and occupational stressors whilst working during the COVID-19 pandemic. However, their anxiety seemed to be buffered by positive organisational support, high levels of resilience and positive coping mechanisms.

#### Anxiety

Across both the consulting practice and hospital staff, anxiety was a considerable stressor during their experience of providing healthcare during the time 1 (*n* = 23) and time 2 (*n* = 9) between March and September, 2020. At time 1, participants reported personal and occupational distress. Personal distress was related to HCW’s health, finances and family balance, whilst occupational distress was the result of the uncertainty experienced by HCWs regarding the changes in organisational routine, processes and the shift from the ‘normal’ (i.e. pre-COVID) way of doing things.

Focussing on personal distress, majority (*n* = 14) of the staff expressed concerns about the health of colleagues, self-contamination and contamination of others, as well as the ability to contain the virus. This is best expressed by Participant 13 (female, 31 years old) who said, ‘It has changed the way we work dramatically. New clothes, masks, constant awareness to germs’. These concerns also led to the participants reporting an increased experience of tension between staff at the site with Participant 23 (male, late-30s) reporting, ‘as more rules are added … it adds tension amongst employees’.

Furthermore, some participants (*n* = 4) commented on their concerns regarding the health of family and loved ones, noting anxiety around whether they would unwillingly transmit the virus to those around them. This was best indicated by Participant 31 (male, 56 years old), who said they were, ‘Nervous about leaving home, as I do not know if I will bring the virus back to my family’.

Lastly, financial concerns arose for some participants (*n* = 4). Participant 12 (male, 55 years old) termed this concern ‘keeping the business sustainable’ as the site had experienced a significant decrease in the number of patients, which was affecting the business’s income and their financial viability. Participant 13 (female, 31 years old) commented, ‘The fear of making turnover and paying salaries were real’.

Occupational stress was evident in many of the sample (*n* = 13) as evidenced in Participant 21’s (female, 53 years old) statement:

‘Nothing is normal anymore. Business is not the same as usual. Life changing moments and a sense of responsibility for your own safety and taking care of your colleagues, customers and doctors to be safe.’ (Participant 21, female, 53 years old)

The new routines, processes and added responsibility seemingly added occupational stress onto staff members.

At time 2, personal stressors, particularly financial viability of the site was still a central point of anxiety (*n* = 7). Financial concerns were a particular concern as fewer patients were being admitted, leading to reduced income for the site. Participants still had to cope with the fear surrounding potential infection, instability of income and the negative impact that COVID-19 was having on patients and colleagues. Thus, staff members were continually challenged and their anxiety surrounding finances and personal safety was often triggered in their jobs. Participant 12 (male, 55 years old) best captured this when they reported that their experience has been, ‘Difficult, adaptation to the way in which things were previously done, more stressful, more risky, dealing with potential infection, loss of income, effect of the virus on patients and colleagues’.

Occupational distress during time 2 was alluded to by majority of the healthcare workers (*n* = 9) when commenting on the operational difficulties related to the working with a skeleton staff, managing with absenteeism, implementation of personal protective protocols and shortage of both PPE and medical supplies. Participant 17 (female, 55 years old) expressed this clearly when she reported the challenge of, ‘Staff shortages when staff off with COVID or isolating – leads to increased load & stress for remaining staff who have to cover for them’. The staff also commented on how changes were necessary, even in the smallest tasks, as new procedural steps needed to be performed. They addressed the difficulties posed by PPE, such as their reading glasses fogging up because of wearing masks for extended periods of time. This led to the feelings of increased challenges to their ability to perform their jobs at the required level.

Furthermore, COVID-19 safety protocols required that the staff have less contact with patients and other staff members, which meant that they had to adjust their daily habits, routines and procedures in their job roles. For instance, Participant 5 (male, 56 years old) commented on how they resented the ‘tedious’ procedures, such as the cleaning. However, other participants (*n* = 5) commented on the screening and prevention protocols that were used to keep the uncertainty at bay. Additionally, increased concern and effort needed to be placed into sourcing supplies and ensuring adequate PPE were available to the staff. These extra tasks placed increased responsibility and workload on staff members, who reported higher workloads because of skeleton staff, absenteeism, longer hours and the need to hold each other accountable for safety behaviours. Participant 7 (female, 54 years old) stated that there was a definite change, ‘Everything you do is different from the norm, being careful and safe at all times. Reminding staff [*to be mindful of germs*]. Supporting staff. Life changing. More strenuous’.

However, implementing and adjusting to these changes appeared to be central to alleviating anxieties and mitigating uncertainty. At time 1, this was evident as Participant 6 (female, 29 years old) noted how the process was ‘challenging initially, but once we had all the correct processes in place, it was a lot easier’. At time 2, participants seemed to demonstrate a stronger acceptance of the ‘new normal’ of operations under COVID-19 and many of the staff (*n* = 9) commenting on the ability of the site to overcome adversity and resume day-to-day activities. However, it is evident that although the new normal was gradually being adjusted to, participants were still feeling anxious because of the difficulty and unpredictability of the environment and the need to keep up with the changes in operations at the site, such as patient screening and increased safety steps taken in operating theatres. Participant 16 (female, 34 years old) wrote, ‘[*it is*] very stressful as we try to find the new normal in a changing world. Uncertainty rules’.

#### Support received

Excellent organisational communication, good personal health and social support were identified as psychosocial buffers mitigating the distress experienced by working during COVID-19 (time 1 *n* = 14, time 2 *n* = 8). Strong communication and support systems were in place at the site, as was evident in both rounds of the survey. Participant 15 (male, 37 years old) at time 1 indicated that they, ‘believe our work place has put in all the measures necessary to make us feel protected’ and Participant 14 (female, 37 years old) at time 2 said, ‘It has been difficult but we’ve had support from our managers and colleagues to get through the worst of it’.

Central to this was the leadership of the doctors and managers, particularly with regard to sharing and accessibility of information and resources. Participants recognised the value of having a part-time consultant coach at the site, because of her offering much in the way of workshops, training and opportunities to debrief during this time. Furthermore, participants reported being grateful for their good health and the social support offered to them by colleagues and co-workers. There was little doubt about the support mechanisms provided by the organisation as exhibited by Participant 13 (female, 31 years old), who commented on the ‘Good communication with staff and coping strategies/availability with our in-house coach’.

The constant support that doctors, managers and colleagues offered in creating a safe and supportive environment was instrumental in helping HCWs to cope with the challenges. Participant 30 (female, mid-50s) indicated, ‘We can speak to anyone who is prepared to listen. Our managers are amazing. Always have time for us’. Such organisational support is likely to function as an important protective buffer for participants. This was particularly evident because of a relatively high number of HCWs who reported that they have no social support other than what they get at work, which was consistent at both time 1 (*n* = 11) and time 2 (*n* = 3) in the study. Thus, it appears that the site provided vital organisational support to many of the staff and was described as an important ‘distraction’ and the only ‘constant’ in many of the participant’s lives, as described by Participant 11 (female, 29 years old) at time 2. Lastly, the physical health of employees remained good, with only a small number in time 1(*n* = 7), time 2 (*n* = 1) and overall (*n* = 8) of participants noting that they had fallen ill or had experienced sleeping difficulties.

## Discussion

### Outline of results

This study investigated mental health experiences of healthcare workers at a private ophthalmic clinic in the Eastern Cape. Overall, the results indicated differences between quantitative results and those obtained through qualitative methods. The quantitative results indicated that HCWs generally experienced generally good psychological health. In addition, from time 1 and 2, it was evident that anxiety, depression and burnout levels decreased, but this could not be tested significantly in the small sample. However, the qualitative responses suggest that HCWs experienced considerable anxiety at both times, particularly at time 1. The HCWs’ ability to cope with the new normal at work is attributed to organisational factors, including organisational support and clear communication, as well as personal factors, including coping strategies and resilience.

#### Anxiety

Based on the quantitative scales, the HCWs presented with good psychological health and seemed to be relatively unaffected by anxiety, depression and burnout. The qualitative responses were examined with a particular focus on work performance, which indicated that some HCWs were in fact experiencing considerable anxiety and experiencing challenges in their daily roles. The qualitative experiences of mental health are consistent with literature that posits that HCWs are vulnerable to anxiety during times of transition and difficulty (Labrague & De los Santos, 2020).

The qualitative results offered further insight, into the very real experience of anxiety that resulted from personal and occupational stressors of working during the COVID-19 pandemic. Personal stressors included concerns related to HCWs’ health, potential of transmission, finances and family balance across time 1 and 2 of the study. This finding is aligned with current research, which suggests that HCWs experience considerable anxiety around becoming infected or unknowingly infecting others (Mo et al., 2020), lack of access to childcare facilities and lack of accurate information on the disease (Shanafelt, Ripp, & Trockel, 2020).

Occupational stressors included operational difficulties, insufficient personnel, change in job roles, procedures and routines and the increased use of PPE. An interesting dilemma was introduced in that increased protective procedural steps and process were implemented to manage much of the uncertainty HCWs were experiencing; however, these were resented and perceived as tedious. According to the literature, new operational procedures and increased use of PPE can be stressful to HCWs and may become detrimental to their performance (Benítez et al., 2020). Challenges adjusting to new procedures and equipment were evident in the qualitative results as participants struggled to adapt to new tasks and processes and their associated challenges, including glasses fogging up because of masks, headaches from wearing PPE and increased responsibility in their roles to prevent infection. However, these procedures and processes were also necessary to protect HCWs and the clinic’s patients. Thus, these results suggest that whilst protective operational processes are absolutely essential, they may be an added source of stress and an obstacle to efficient job performance (Benítez et al., 2020). Although participants reported increased obstacles to their performance, there was no considerable impact on their job performance.

#### Support structures

Despite the existence of considerable stressors identified in the qualitative responses, HCWs did not display high levels of stress, anxiety and burnout in the quantitative measures. This finding was not in line with expectations, but may be attributed to the various forms of support that this sample of HCWs received.

### Communication and social support

One form of support available to this sample included positive leadership by management, who focussed on communication and support of the HCWs. Transparent and honest communication of the realities of the situation, including the finances, and provision of informational resources seemed to play a positive role in making HCWs feel informed. Accessibility to a coach and the managers was essential in making participants feel that their concerns were heard. This seems to have been a constant theme within COVID-19 research, as the importance of communication with HCWs regarding challenges, uncertainties and strategies seems to play a major role in helping them cope with the unprecedented circumstances (Walton et al., 2020).

Organisational support seemed to be particularly pertinent, as this influences the provision of resources, reinforcement, encouragement and communication with employees (Labrague et al., 2018). Organisational support plays an important role ensuring positive outcomes in HCWs’ work performance and patient satisfaction and reduces the impact of anxiety in hazardous circumstances (Jung, Jung, Lee, & Kim, 2020; Labrague et al., 2018). Thus, two organisational factors, namely, open and honest communication and accessible support, are identified to potentially be protective of HCW’s mental health and the subsequent job performance. Firstly, communication that is open and honest is important in encouraging the acknowledgement of difficult emotions, such as fear and anger (Walton et al., 2020). Secondly, the formalisation of channels for communication is essential in ensuring that HCWs have a means to seek the support they need (Walton et al., 2020). Organisations can offer a form of social support to HCWs through having formalised communication channels, which may positively impact their work performance (Saeng et al., 2020).

### Positive coping strategies

Participants drew on positive coping strategies at both points in this study. At both time 1 and 2, active coping was positively endorsed by HCWs, but this was the only common coping strategy. At time 1, participants relied on the coping styles of planning and religion, whilst at time 2, participants reported relying on coping styles of acceptance and positive framing. This finding is interesting, as participants relied heavily on active coping, but also adopted new strategies in different phases of the pandemic. For instance, at time 1, uncertainty ‘ruled’ and participants relied on religion and planning. However, at time 2 when there was slightly more certainty, participants seemed to accept reality and try to see it in a positive manner. This can inform future coping strategy interventions to target these coping strategies at different phases of a crisis in future (Zhu et al., 2020). Healthcare workers did not seem to rely on unhelpful coping mechanisms, such as behavioural disengagement and substance use.

This finding is consistent with previous literature, as positive coping strategies have been associated with decreased psychological distress, anxiety and stress amongst HCWs (Zhu et al., 2020). Indeed, a potential relationship may exist between coping strategies and social support as positive coping is shown to strengthen the positive effect of social support on anxiety in HCWs in China (Zhu et al., 2020). If there was not enough social support, individuals were likely to adopt poor coping styles. Thus, because of the prevalence of organisational support, HCWs may have adopted better coping strategies against anxiety, depression and burnout (Zhu et al., 2020). This is an avenue for further research.

### Resilience

Participants displayed good levels of resilience at both measurement opportunities. In fact, participant’s resilience increased across the two measurements. Research suggests that resilience, or an individual’s capacity to ‘bounce back’ from stress (Hart, Brannan, & De Chesnay, 2014), helps HCWs cope with the stress caused by the pandemic and adapt successfully to changed circumstances (Cooper, Phelps, Ng, & Forbes, 2020). Thus, strengthening HCWs’ levels of resilience may help enhance psychological health, mental well-being and their capability to continue to perform at work (Labrague & De los Santos, 2020).

#### Practical implications

Healthcare workers have been exposed to significant occupational and situational stress whilst working during the COVID-19 pandemic. It is suggested that organisational and individual interventions play a role in helping healthcare workers manage this stress. In this study, the institution, leadership and management played a key role in creating an environment where HCWs felt safe and supported, especially in times of crisis. This finding indicates that these stakeholders are core in protecting HCWs and ensuring a productive workplace (Di Tella, Romeo, Benfante, & Castelli, 2020; Robertson et al., 2020; Walton et al., 2020). Based on this research, healthcare as well as other institutions should ensure that leadership maintain open and honest communication with their employees about the realities of the current situation, how it may affect them and steps that are being taken to mitigate risk to the business and employee. These steps ensure that employees feel supported and informed regarding the current context and the likely implications thereof (Saeng et al., 2020). Additionally, organisations must ensure that the needed support is accessible through formalising channels for communication and ensuring awareness of this, so that the employees have a means to seek the support they need (Saeng et al., 2020; Walton et al., 2020). These interventions may be protective of the employee’s mental health and subsequently, their job performance.

On an individual level, HCWs should undergo training or receive support in developing resilience and positive coping strategies as these seem to act as a buffer against anxiety, depression and burnout. For instance, it may be useful to encourage different coping mechanisms at different stages of a threatening situation to aid employees in coping and responding optimally to the situation. Developing positive coping strategies and resilience could be supported by short-term interventions or regular check ins with colleagues and management to maintain accountability and a sense of a support structure (Labrague et al., 2018).

### Limitations

Whilst this study offered useful insight into HCWs real lived experiences of working during COVID-19, it must be acknowledged that this is a small sample of HCWs who worked in a private consulting and hospital context. Of the 50 available staff members, only 62% (*n* = 31) responded at time 1 and 30% (*n* = 15) responded at time 2. Thus, HCWs in more direct contact with COVID-19 and who were in greater distress may not have responded. Furthermore, because of the decreased response rate at time 2, a comparative analysis could not be conducted.

## Conclusion

This study highlights the importance of organisational support and indicates why protecting HCWs’ well-being is a crucial tool in ensuring an effective and sustainable response to the public health emergency South Africa is currently facing. In this study, HCWs were able to maintain a normal level of mental health and although they faced challenges, their performance did not seem to be significantly impacted. Personal resilience, positive coping strategies and organisational support were identified as vital factors protecting against anxiety, depression and burnout in HCWs. This study indicates how the accessible organisational support and communication helped the staff members to draw on positive coping skills and resilience so that they did not exhibit unhealthy levels of anxiety, depression or burnout and were likely to have continued to work at an appropriate level. This study can be used as a case study for other medical facilities, or any other institution, to follow in protecting their employees’ mental health and ability to perform their roles, which ultimately protects their business viability. Furthermore, if used as a case study, perhaps this represents an opportunity to build a more compassionate and sustainable healthcare system in South Africa.
